# Risk of breast cancer in the UK biobank female cohort and its relationship to anthropometric and reproductive factors

**DOI:** 10.1371/journal.pone.0201097

**Published:** 2018-07-26

**Authors:** Kawthar Al-Ajmi, Artitaya Lophatananon, William Ollier, Kenneth R. Muir

**Affiliations:** Division of Population Health, Health Services Research and Primary Care, School of Health Sciences, Faculty of Biology, Medicine and Health, University of Manchester, Manchester, United Kingdom; McMaster University, CANADA

## Abstract

**Background:**

Anthropometric and reproductive factors have been reported as being established risk factors for breast cancer (BC). This study explores the contribution of anthropometric and reproductive factors in UK females developing BC in a large longitudinal cohort.

**Methods:**

Data from the UK Biobank prospective study of 273,467 UK females were analyzed. Relative risks (RRs) and 95% confidence intervals (CIs) for each factor were adjusted for age, family history of BC and deprivation score. The analyses were stratified by the menopausal status.

**Results:**

Over the 9 years of follow up the total number of BC cases were 14,231 with 3,378 (23.7%) incident cases with an incidence rate of 2.09 per 1000 person-years. In pre-menopausal, increase in age, height, having low BMI, low waist to hip ratio, first degree family history of BC, early menarche age, nulliparous, late age at first live birth, high reproductive interval index, and long contraceptive use duration were all significantly associated with an increased BC risk. In post-menopausal, getting older, being taller, having high BMI, first degree BC family history, nulliparous, late age at first live birth, and high reproductive interval index were all significantly associated with an increased risk of BC. The population attributable fraction (PAF) suggested that an early first live birth, lower reproductive interval index and increased number of children can contribute to BC risk reduction up to 50%.

**Conclusions:**

This study utilizes the UK Biobank study to confirm associations between anthropometric and reproductive factors and the risk of breast cancer development. Result of attributable fraction of risk contributed by each risk factor suggested that lifetime risk of BC can be reduced by controlling weight, reassessing individual approaches to the timing of childbirth and options for contraception and considering early screening for women with family history in the first degree relative.

## Introduction

Breast cancer is the most common cancer in females, globally accounting for 23% of all new female cancers [1–4]. In the UK, BC accounts for 15% of all newly diagnosed cancer cases in the population regardless of gender [5]. Global variations in BC incidences arise mainly from the availability of early detection and treatment facilities; however other factors may also affect this variation. Factors such as population structure (age, ethnicity, and race), life expectancy, environment, lifestyle, prevalence of risk factors, health insurance status, availability of new treatments, and pathology can enhance this variation [6]. Several risk factors have been reported in the literature. Reproductive risk factors including, early age at menarche, late menopause age, late age at first birth, low parity, hormonal replacement therapy usage, contraceptive use, hysterectomy and bilateral oophorectomy have all been identified as conferring risk for developing BC [7, 8]. Another major factor for increasing BC incidences is the accumulated effect of anthropometric factors. Increased height, weight, hip circumference, waist circumference, body mass index (BMI), and waist to hip ratio (WHR) have been reported as increasing BC risk depending on the menopausal status of women [9]. Given the unique opportunity the UK biobank [2] project offers for assessing a wide range of disease risk factors in a large longitudinal cohort, we have measured the effect of anthropometric and reproductive factors on BC risk. This study is the first study to explore the relationships of risk factors and breast cancer in the UK Biobank initiative. This landmark national cohort provides an important dataset based on half a million UK residents. The recruitment was undertaken and 22 regional centers to seek distributed population coverage across the UK. The cohort also has broad-scale genotyping performed which will allow further investigations of the possible combined effects of the genetic and the epidemiological risk factors reported in this paper.

## Materials and methods

### Study population and study design

UK Biobank is a national-based health project that aims to improve the diagnosis, treatment, and prevention of diseases such as cancers, diabetes, stroke, heart disease, osteoporosis, arthritis, eye diseases, dementia and depression [2]. A total of 502,650 participants aged between 39 to 71 years were enrolled in the study between 2006 and 2010 and they continue to be clinically followed up. Details can be found at http://www.ukbiobank.ac.uk/. In addition to the collection of biological samples (blood, saliva and urine), health, demographic and anthropometric data were collected in 22 UK assessment facilities across England, Wales and Scotland. Detailed physical / physiological measurements were further supported by the administration of questionnaires and eye examination. Many participants completed additional detailed questionnaires on work history, diet, and cognitive function. Anonymized data are now available to researchers across the world [2, 3]. Our study acquired data on the female cohort (273,467 female participants) from UK Biobank. The UK Biobank female cohort had a mean follow up time of 6.9 years (at 2016). Data on exposures were defined prior to the development of BC in cases or prior to the first assessment date in controls.

#### Defining breast cancer cases and controls

BC was defined as a malignant neoplasm of the breast. The UK Biobank database contained record of all cancers including their subtype occurring either before or after participant enrollment using the International Classification of Diseases (ICD10, ICD9) and their self-reported data. Details of codes used to identify BC cases are summarized in S1 Table.

#### Breast cancer cases

In the database, each participant had 9 follow-up time point records for ICD10, 11 follow-up time point records for ICD9 and 9 follow-up time point records for self-reported status of cancer. The case-control groups were identified by utilizing all these three data sources. The codes for BC are presented in S1 Table. Cases were characterized as incident or prevalent using ‘age or date when they attended the center’ and ‘age when first reported BC cancer’. With cases defined by ICD10 and ICD9, if their ‘attending age’ was greater than ‘cancer diagnosis age’ then this was considered as a prevalent case. Subjects were considered to be incident cases if their ‘attending age’ was less than their ‘cancer diagnosis age’. For self-reported cases, the same criteria were applied. Age when first attended the assessment center was compared with the interpolated age of the participant when cancer was first diagnosed. To combine and classify the type of cases from 3 different sources, we applied the following criteria:

1. If the BC cases appeared as being incident using any of these three identification methods then the cases were deemed to be incident cases.

2. Prevalent cases were defined using combination of rules a) only if the participant has been identified as a prevalent case by any of the three methods and b) none of these methods define the same participant as being an incident case.

In total, there were 14,231 BC cases with 3,378 being incident cases and 10,853 prevalent cases.

#### Controls

Female participants were defined as controls if they had no record of cancer, *in-situ* carcinoma or an undefined neoplasm (232,476 controls).

#### Exclusion criteria

In the case group, we excluded 10,853 (3.97%) prevalent BC cases. In the control group, participants were excluded due to following reasons; other type of cancers (23,540), breast *in situ* carcinoma (636), other *in situ* carcinomas (2,463) and unknown neoplasm (121).

#### Exposures

Reproductive variables included menarche age, menopause age, menopausal status, parity (yes/no), number of children, age at first live birth, pregnancy history, pregnancy termination and number of terminations, reproductive interval index (difference between menarche age and age at first birth), history of oral contraceptive (OC) use and its duration, and history of hormonal replacement therapy (HRT) use and its duration. Anthropometric variables included BMI, waist to hip ratio (WHR) and height (sitting and standing).

### Statistical analysis

To assess associations between exposures and BC risk in the cohort, we computed relative risk (RR) and 95% confident intervals (95% C.I.) using a binomial generalised linear regression model. Regression analyses were performed for each independent variable and were adjusted for age, family history of BC in first degree relatives, and deprivation score. The independent variables list and description are presented in S2 Table.

All analyses were stratified by menopausal status: pre- and post-menopausal. The criteria for pre-menopausal were females aged ≤ 55 years old (according to the NHS the menopause age in the UK is between 40 to 55 years [10]) who reported that they still had periods and did not report a history of hysterectomy or bilateral oophorectomy, and menarche age ≥ 7 years old (the menarche age in the UK ranges from 7 to 20 years [11]). Post-menopausal females were defined as those who reported no longer having periods and did not report a history of hysterectomy or bilateral oophorectomy and their menopause age ≥ 40 years old. These criteria were employed to minimise inclusion of both pre-mature and the medically induced pre- or post-menopausal women. After further application of criteria, 61,903 participants were in pre-menopause group and 133,704 participants were in post-menopause group.

To compute BC incidence within the cohort, we used the STATA *stptime* command to obtain the overall person-time of observation and disease incidence rate. To calculate time for each participant, we subtracted the endpoint (either the date of cancer diagnosis or the end of the follow-up—January 1^st^, 2016) with the date of study enrolment. Incidence rates were estimated for the whole cohort and pre- and post-menopausal separately. Moreover, population attributable fractions (PAF) were calculated using the *punaf* command [12] where the fraction was estimated compared to whole cohort and compared to the most significant subgroup associated with the BC. This was done to estimate how much risk could be eliminated by controlling that risk factor in both groups.

All statistical analysis was performed using STATA MP 14.1 software for Windows [13]. Results with 95% confident intervals not including 1 were considered as being statistically significant.

## Results

**Fig 1:**
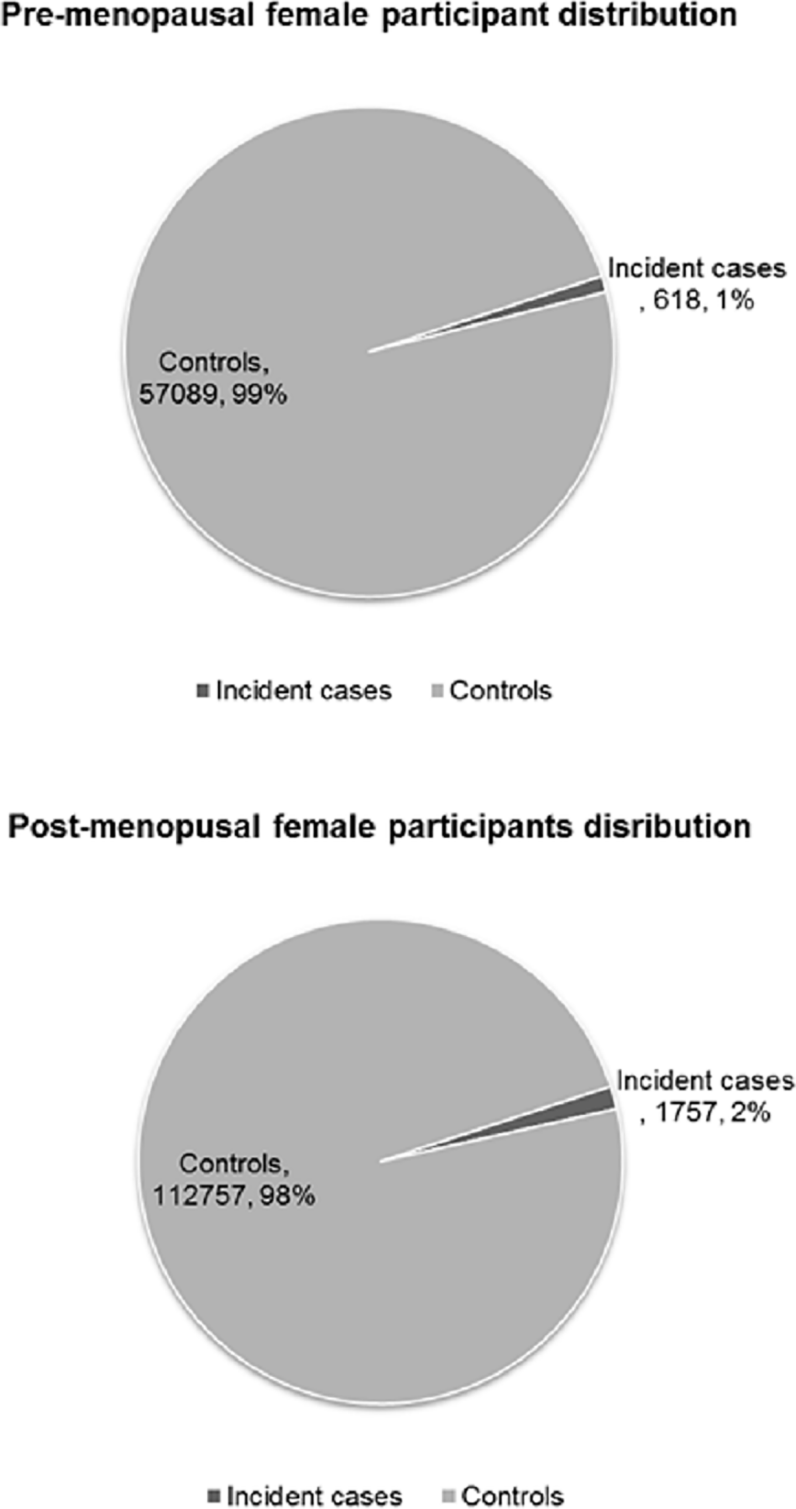
UK biobank data distribution based on menopausal status.

The UK biobank female cohort consisted of 273,476 female participants with a mean age of 56.3 years (SD ±8.00). The follow up time was 9.8 years up to January 2016 where the database was frozen for this analysis. The total number of BC cases was 14,231 with 3,378 (1.24%) incident cases and 10,853 (3.97%) prevalent cases. The total number of controls was 232,476 (85.01%). The remaining participants were either females with other cancer 23,540 (8.61%) or with breast *in situ* carcinoma 636 (0.23%), or other *in situ* carcinoma 2,463 (0.90%) or unknown neoplasm 121 (0.04%). A total of 3,162 (93.60%) of incident cases were identified by ICD10 and the rest 216 (6.40%) were identified by self-reporting. All the BC cases identified by ICD9 were solely prevalent cases. When further applying criteria for menopause status, the total number of pre-menopausal females was 61,903 (31.65%) and post-menopausal was 133,704 (68.35%). Out of the total pre-menopausal females, 618 (1.07%) were incident cases and 57,089 (98.93%) were controls. For post-menopausal females, 1,757 (1.53%) were incident cases and 112,757 (98.47%) were controls (Fig 1). The BC incidence rate of the whole cohort was 2.09 per 1000 person-years. The pre-menopause BC incidence rate was 1.55 per 1000 person-years and the post-menopause BC incidence rate was 2.24 per 1000 person-years. The incidence rate ratio between the pre- and post-menopausal females is 1.45 with 95% CI 1.32–1.59.

Comparisons of mean values of age, deprivation score, anthropometric and reproductive variables (all continuous variables) of the participants conditioned on the menopausal status are summarised in Table 1. In both pre- and post-menopause groups, cases were older than controls and the mean age differences were statistically significant (*Student’s t-test p-values<0*.*05*). Results using the Townsend deprivation score showed that case’s mean score were significantly lower than control mean score in both pre- and post-menopause females (*Student’s t-test p-values* < 0.05).

**Table 1:** Mean comparisons between cases and controls in pre- and post-menopause status.

	Pre-menopausal				Post-menopausal			
Variables	No. (cases/controls)	Case’s mean	Control’s mean	P-value*	No. (cases/controls)	Case’s mean	Control’s mean	P-value*
Age (year)	(618/ 57,089)	46.43	45.83	<0.001	(1,757 /112,757)	60.67	59.76	<0.001
Deprivation score	(618/56,999)	-1.49	-1.09	0.001	(1,755 /112,639)	-1.72	-1.48	0.006
Body shape measures								
BMI (kg/m2)	(612/ 56,847)	25.95	26.43	0.026	(1,750/112,270)	27.45	27.01	<0.001
Waist Circumference (cm)	(613 /56,890)	80.97	82.23	0.012	(1,752/112,426)	86.03	84.78	<0.001
Hip Circumference (cm)	(613 /56,889)	102.16	102.51	0.408	(1,752/112,423)	104.32	103.12	<0.001
Waist to Hip ratio	(613 /56,883)	0.79	0.80	<0.001	(1,752/112,416)	0.82	0.82	0.114
Standing Height (cm)	(612 /56,896)	164.70	164.04	0.011	(1,751/112,391)	162.61	161.91	<0.001
Sitting height (cm)	(603 /56,406)	87.86	87.54	0.031	(1,724/111,654)	86.36	86.03	<0.001
Reproductive factors measures								
Menarche age (year)	(605 /55,286)	12.95	13.05	0.105	(1,727/110,214)	12.93	12.98	0. 178
Menopause age (year)	N/A				(1,757/112,757)	50.85	50.58	0.007
Number of live births	(618 /57,053)	1.49	1.57	0.095	(1,754/112,685)	1.77	1.88	<0.001
Age at first birth (year)	(336 /33,071)	27.70	27.03	0.015	(1,171/79,421)	25.46	25.30	0.231
Number of Pregnancy termination	(221 / 20,149)	0.61	0.69	0.127	(529/34,166)	0.47	0.52	0.140
Reproductive interval index (year)	(521/47,237)	14.66	13.93	0.011	(1,483 /96,718)	12.50	12.29	0.131
Contraceptive use duration (year)	(519/ 50,012	11.62	9.99	<0.001	(1,610/ 102,760)	7.51	7.68	0.386
HRT duration (year)	(609/56,210)	0.05	0.03	0.200	(1,553/ 102,786)	2.25	1.92	<0.001
Total	618 / 57,089				1,757 / 112,757			

*Student’s t-test

For anthropometric variables, in the pre-menopausal group, the mean values of standing and sitting height in cases were higher as compared to controls (*Student’s t-test p-values<0*.*05*). On the other hand, mean values of BMI, waist circumference and waist to hip ratio were significantly lower in cases as compared with controls (*Student’s t-test p-values<0*.*05*). In the post-menopause case group, the mean values of standing and sitting height, BMI, waist circumference, and hip circumferences were higher when compared with controls (*Student’s t-test p-values<0*.*05*).

Analysis of reproductive factors in pre-menopause case group, showed higher mean values of age at first birth, reproductive interval index, and contraceptive use duration as compared with controls (*Student’s t-test p values <0*.*05*). In addition, among the post-menopausal group, mean values of menopause age and duration of HRT use were significantly higher in cases compared with controls. In contrast, mean values of number of live births were lower in cases as compared to controls in post-menopausal females.

Relative risks (RRs) of the key characteristics and anthropometric measures of pre- and post-menopausal females are illustrated in Table 2. For both pre-and post-menopausal females, age as a continuous variable showed a slight increased risk of developing BC (RR = 1.05, 95%CI; 1.02–1.07) and RR = 1.03, 95%CI; 1.02–1.04, respectively). Results of Townsend deprivation score showed a decreased risk of BC associated with increased deprivation score (more deprived) among both pre- (RR = 0.96, 95%CI; 0.94–0.99) and post-menopausal (RR = 0.97, 95%CI; 0.96–0.99) females.

**Table 2:** Relative risk of key characteristics and anthropometric factors in pre- and post- menopausal females.

Menopausal status	Pre-menopausal					Post-menopausal				
Variables	Number of cases/controls	RR	P-value	LCL	UCL	Number of cases/controls	RR	P-value	LCL	UCL
Age in years (Continuous) *	618/ 57,089	1.046	<0.001	1.024	1.069	1,757 /112,757	1.033	<0.001	1.024	1.042
Deprivation score (Continuous) **	618/56,999	0.962	0.004	0.937	0.988	1,755 /112,639	0.973	0.001	0.957	0.990
Family history ***										
No	520/ 51,547	Ref				1,458/ 99,998	Ref			
Yes	97/ 5,326	1.770	<0.001	1.427	2.194	290/ 12,367	1.582	<0.001	1.397	1.792
Mother BC history ***										
No mother BC history	532/ 51,750	Ref				1,529/ 102,184	Ref			
Mother BC history	78/ 4,360	1.724	<0.001	1.362	2.181	192/ 8,145	1.569	<0.001	1.353	1.820
Sibling BC history ***										
No sibling BC history	579/ 54,125	Ref				1,553 / 103,570	Ref			
Sibling BC history	23/ 1,108	1.823	0.004	1.206	2.756	120/ 4,782	1.613	<0.001	1.343	1.938
Family history- Combined***										
No family history at all	520/ 51,547	Ref				1,458/ 99,998	Ref			
Mother or Sister BC history	93/ 5,184	1.756	<0.001	1.408	2.190	268/11,807	1.540	<0.001	1.351	1.754
Mother and Sister BC history	4/142	2.592	0.054	0.982	6.837	22/560	2.594	<0.001	1.717	3.920
BMI in kg/m^2^ (Continuous)	612/ 56,847	0.983	0.041	0.968	0.999	1,750/112,270	1.018	<0.001	1.009	1.027
BMI–categorical										
BMI—Healthy (18.5–24.9)	326/26,983	Ref				626/44,215	Ref			
BMI—Overweight (25–29.9)	186/18,319	0.839	0.055	0.701	1.004	681/42,624	1.102	0.078	0.989	1.228
BMI—Obese (> = 30)	100/11,545	0.733	0.007	0.586	0.918	443/25,431	1.241	0.001	1.098	1.401
Waist to Hip (Continuous)	613 /56,883	0.131	0.001	0.038	0.446	1,752/112,416	1.520	0.226	0.772	2.994
Waist to Hip–categorical										
Waist to Hip—Low (< = 0.80)	362/30,170	Ref				678/45,184	Ref			
Waist to Hip—Moderate (0.81–0.85)	139/13,993	0.829	0.060	0.682	1.008	475/30,741	1.010	0.869	0.898	1.135
Waist to Hip—High (>0.85)	112/12,720	0.744	0.006	0.602	0.920	599/36,491	1.073	0.213	0.961	1.198
Sitting Height in cm (Continuous)	603 /56,406	1.023	0.041	1.001	1.046	1,724/111,654	1.032	<0.001	1.019	1.046
Standing Height in cm (Continuous)	612 /56,896	1.017	0.010	1.004	1.030	1,751/112,391	1.021	<0.001	1.013	1.029
Standing Height in cm–categorical										
Below mean ± SD (150.20–156.06 cm)	57/6,447	Ref				285/21,259	Ref			
Within mean ± SD (159.21–165.71 cm)	388/37,137	1.181	0.243	0.893	1.562	1,153/ 75,173	1.168	0.019	1.025	1.330
Above mean ± SD (169.02–175.00 cm)	167/13,314	1.429	0.021	1.057	1.933	313/ 15,964	1.533	<0.001	1.305	1.802

All adjusted for age + Family history of BC + deprivation score

*adjusted for deprivation score only

** no adjustment

***Adjusted for age + deprivation score

Family history of BC is a well-defined risk factor for BC. The strength of this risk factor varies according to the number and relationship of the affected family members. Females who reported having had a family history of BC were at increased risk for developing BC in both pre- and post-menopausal females with (RR = 1.77, 95%CI; 1.43–2.19) and (RR = 1.58, 95%CI; 1.40–1.79), respectively. Both pre- and post-menopause subjects with their siblings affected with BC were at increased risk of 82% (pre-menopause) and 61% (post-menopause) respectively. Similar results were also seen in subjects who reported only their mother affected with BC with increased risk of 72% in pre- and 57% in post-menopausal women. All of these significant associations were stronger among pre-menopausal compared to post-menopausal women. In the post-menopause group, subjects with both mother and sibling affected with BC were almost at three-fold increase BC risk (RR = 2.59, 95%CI; 1.72–3.92). Despite a similar relative risk estimate, no association was reported in pre-menopause group.

For anthropometric exposures treated as being continuous variables, increasing BMI (RR = 0.98, 95%CI; 0.97–1.00), and waist to hip ratio (RR = 0.13, 95%CI; 0.04–0.45) were associated with reduced BC risk among the pre-menopause group. The WHR as a categorical variable (low as reference group, moderate and high) showed significant risk reduction only in the high WHR group (RR = 0.74 with 95%CI; 0.60–0.92). BMI as a categorical variable showed that obese women with a BMI ≥30 had 26.7% decreased BC risk compared to women with normal range BMI. For height, per 1 cm of increased height (cm), BC risk was increased by 2%. Height as a categorical variable showed that women in the tallest group (height ranges from 168.8 to 199 cm) had their BC risk increased by 43% compared to shorter females with height ranges from 152.20 to 156.06 cm.

In post-menopausal women, increasing BMI, standing height and sitting height were associated with a slight increased risk of BC of 2%, 2% and 3%, respectively. BMI as a categorical variable showed that obese subjects had 24.1% increased risk for BC (RR = 1.24, 95%CI; 1.10–1.40) when compared to the normal BMI group. For height treated as a categorical variable, results suggested that the tallest group (height ranges from 168.8 to 199 cm, mean = 172.0) were at 53% increased risk of BC (RR = 1.53, 95%CI; 1.31–1.80) when compared to the reference group (height ranges from 100 to 156 cm, mean = 153.1).

### Reproductive factors and breast cancer

RRs for the reproductive factors and BC risk are presented in Table 3. For the pre-menopause group, menarche age as continuous variable showed a slight risk reduction (RR = 0.95, 95%CI; 0.90–1.00). When menarche age was grouped into >13 years old (as a reference group) versus ≤13 years old, a moderate increased risk was observed (RR = 1.23, 95% CI; 1.04–1.45). For the post-menopause group, age at menarche did not show any significant association with BC risk (confidence interval value included 1).

**Table 3:** Relative risks of the reproductive factors based on the menopausal status.

Menopausal status		Pre-menopausal					Post-menopausal			
Variables	No. cases/controls	RR	P-value	LCL	UCL	No. cases/controls	RR	P-value	LCL	UCL
Menarche age in years (Continuous)*	605 /55,286	0.948	0.042	0.900	0.998	1,727/110,214)	0.987	0.388	0.958	1.017
Menarche age–categorical										
Menarche age (>13)	198/ 20,785	Ref				625 /40,534	Ref			
Menarche age (≤13)	407/ 34,501	1.228	0.017	1.037	1.454	1,102/69,680	1.029	0.569	0.933	1.134
Menopause age in years (Continuous)*	Not applicable					1,757/112,757	1.006	0.284	0.995	1.018
Parity										
No	188/15,024	Ref				326/18,855	Ref			
Yes	430/42,029	0.764	0.002	0.643	0.908	1,428/93,830	0.821	0.001	0.728	0.926
Number of births (Continuous)	618 /57,053	0.925	0.024	0.864	0.990	1,754/112,685	0.899	<0.001	0.863	0.937
First live birth age in years (Continuous)	336 /33,071	1.022	0.055	1.000	1.045	1,171/79,421	1.010	0.142	0.997	1.023
First live birth age–categorical										
First live birth age (<20)	12/2,422	Ref				97/7,330	Ref			
First live birth age (20–24)	74/7,873	1.719	0.082	0.933	3.168	369/27,992	0.966	0.763	0.773	1.207
First live birth age (25–29)	138/12,625	1.882	0.038	1.036	3.417	492/31,181	1.091	0.435	0.876	1.360
First live birth age (≥30)	112/10,151	1.938	0.031	1.062	3.539	186/12,918	1.055	0.669	0.825	1.350
pregnancy termination										
No	117/9,544	Ref				321/ 19,771	Ref			
Yes	104/10,605	0.835	0.181	0.641	1.088	208/14,395	0.981	0.834	0.823	1.171
Pregnancy termination number (Continuous)	221 / 20,149	0.898	0.232	0.753	1.071	529/34,166)	0.973	0.673	0.858	1.104
Reproductive interval index in years (Continuous)	521/47,237	1.003	0.002	1.001	1.005	1,483 /96,718	1.003	<0.001	1.001	1.004
Reproductive interval index–categorical										
Low index (≤12)	109/12,673	Ref				585/41,334	Ref			
Moderate index (12.01–16)	98/9,499	1.146	0.329	0.872	1.506	359/22,601	1.128	0.073	0.989	1.287
High index (>16.01)	126/10,041	1.421	0.008	1.098	1.838	213/13,928	1.130	0.128	0.965	1.323
No children	188/15,024	1.530	<0.001	1.208	1.937	326/18,855	1.333	<0.001	1.163	1.528
Contraceptive use										
No	53/ 6,297	Ref				366/23,896	Ref			
Yes	565/50,646	1.261	0.106	0.952	1.670	1,389/88,638	1.124	0.053	0.998	1.265
Contraceptive duration in years (Continuous)	519/ 50,012	1.024	<0.001	1.013	1.034	1,610/ 102,760	1.003	0.319	0.997	1.010
HRT use										
No	599/ 55,336	Ref				943 /65,669	Ref			
Yes	18/1,565	0.945	0.813	0.590	1.513	811/46,830	1.141	0.006	1.038	1.255
HRT duration in years (Continuous)	609/56,210	1.063	0.298	0.947	1.193	1,553/ 102,786	1.013	0.054	1.000	1.025
Mammogram history										
No	359 /37,546	Ref				50/5,408	Ref			
Yes	285/19,341	1.190	0.054	0.997	1.420	1,706/107,289	1.260	0.120	0.942	1.686

All adjusted for age, family history of BC and deprivation score

* adjusted more for BMI.

Parous women were at reduced BC risk in both pre- (RR = 0.76, 95% CI; 0.64–0.91) and post-menopausal women (RR = 0.82, 95% CI; 0.73–0.93) when compared to nulliparous women. The ‘number of children’ when treated as a continuous variable showed moderate decreased BC risk (pre-menopause group RR = 0.93, 95% CI; 0.86–0.99 and post-menopause group (RR = 0.90, 95% CI; 0.86–0.94). In contrast, increasing maternal age at live birth showed very slight increased BC risk in both pre- (2%) and post-menopausal women (1%). Further analysis was carried out in parous women to explore the association of age at live birth and BC risk. Age at first live birth as categorical variable (< 20 years old as the reference group, 20–24, 25–29, and ≥30 years old) showed that among pre-menopausal females, BC risk was almost double when they reported having had their first child at age ≥30 years old and at age 25–29 years as compared to women who reported having their first baby at age <20 years old (RR 1.94; 95% CI, 1.06–3.54 and RR = 1.88 with 95% CI; 1.04–3.42, respectively). This effect was not seen in post-menopausal females (all 95% CI values included 1). Both pregnancy termination history (ever versus none) and number of terminations were not significantly associated with BC development in both pre- and post-menopausal females (all 95% CI values included 1).

The Reproductive Interval Index (the difference between age at first child and the age of menarche) based on the interquartile range of the control group (low as reference group, moderate, high, and no children) only showed statistically significant increased risk in ‘high’ (RR = 1.42, 95% CI; 1.10–1.84) and ‘no children’ groups (RR = 1.53, 95% CI; 1.21–1.94) in pre-menopausal females. In post-menopausal group, only females reporting no children showed an increased risk of BC (RR = 1.33, 95% CI; 1.16–1.53) when compared to the low index group.

History of oral contraceptive (OC) pills used showed no association with BC risk in both pre- and post-menopause groups. Within the OC use group, however, OC duration showed a slight increased BC risk in pre-menopause women of 2% but not in post-menopausal women. Hormone replacement therapy (HRT) was not associated with risk of BC in pre-menopause UK females. In the post-menopause group, women who reported using HRT were at moderate significant increased risk (RR = 1.14, 95%CI; 1.04–1.26).

Women in both pre- and post-menopausal groups who reported having had mammograms were at increased risk of BC of 19% and 26%, respectively.

PAF were calculated for the modifiable risk factors only based on the menopause status (Table 4). Two fractions were estimated; the PAF among the studied population and the PAF among the sub-population (the exposed significant group) to evaluate how many cases could be avoided if a particular factor was eliminated. Among pre-menopausal females these modifiable factors were the strongest in reducing the BC risk. Giving birth at age <30 can eliminate about 44.6% of the BC cases in general population, and about 48.4% among females who had first children at age ≥30years old and about 46.9% of cases among females who had first children at age 25–29. Followed by low reproductive interval index with about 34.6% of BC cases can be eliminated among null-parous females and about 29.6% of BC cases can be eliminated among females with high index (>16.01). Being parous can eliminate only 9.2% of the cases without taking into consideration the number of children they gave birth to. Finally, having BMI ≥30 and WTH >0.85 can eliminate 70% and 66.2% of the cases among pre-menopausal women, respectively.

**Table 4:** Population attributable fraction (PAF) among modifiable breast cancer risk factors according to the menopausal status.

Variables	Pre-menopausal		Post-menopausal	
	PAF in population	PAF in subpopulation group	PAF in population	PAF in subpopulation group
BMI				
BMI—Healthy (18.5–24.9)	Ref			
BMI—Obese (> = 30)	-0.091	-0.707	0.083	0.194
Waist to Hip ratio				
Waist to Hip—Low (< = 0.80)	Ref			
Waist to Hip—High (>0.85)	-0.080	-0.662	*NS*	*NS*
Parity (Yes/No)				
Yes	Ref			
No	0.072	0.092	0.033	0.179
Number of births				
None	Ref			
More than one child	0.088	0.247	0.046	0.211
First live birth age				
First live birth age (<20)	Ref			
First live birth age (25–29)	*NS*	0.469	*NS*	*NS*
First live birth age (≥30)	0.446	0.484	*NS*	*NS*
Reproductive interval index				
Low index (≤12)	Ref			
High index (>16.01)	0.149	0.296	*NS*	*NS*
No children	0.223	0.346	0.089	0.250
HRT use (No /Yes)				
No	Ref			
Yes	*NS*	*NS*	0.058	0.125

Among post-menopausal women; reducing BMI <30 can eliminate 8.3% among general population and 19.4% among obese females; being parous can eliminate 17.9% among null parous females; having more than one child can eliminate 21.1% % among females with <1 child; not using HRT can eliminate 12.5% of cancer cases among users.

The most effective preventative factors identified were giving birth at earlier age, having more than one child, reducing the reproductive interval index, and reducing weight.

A summary for the significant factors associated with development of BC among UK females is presented in S3 Table.

## Discussion

This study explores the effect of anthropometric and reproductive factors on risk of developing BC in the UK Biobank female cohort. The BC incidence rate in the pre-menopause group was 1.55 per 1000 person-years and 2.24 per 1000 person-years in the post-menopause group. McPherson *et al* reported a similar finding that in every 1000 UK women over 50 years old, two females will be diagnosed with BC [14] which suggests that UK biobank is a representative cohort of the UK female population.

Findings from previous studies suggested that differences in risk factors and incidences of BC were based on the menopausal status [4, 5, 15]. Some of the risk factors were common across pre- and post-menopause groups while other factors showed different effects. We therefore stratified all the analyses by menopausal status.

### Age

For both pre- and post-menopausal groups, age is associated with increasing risk of developing BC. Age is a well-established risk factor for BC [16]. BC incidence increases with age during the reproductive years by the double in every 10 years up until the menopause [5, 15]. A potential explanation could be cells becoming more susceptible to environmental carcinogens and modification in the biological ageing which stimulates or allows tumour growth and metastasis [17].

### Family history

Family history of BC is also a well-established risk factor. Our findings suggested that females with a first degree relative (sibling or mother) affected with BC were at high risk of developing BC. Regardless of menopause status, the estimated risks were higher in females who reported only their sibling(s) affected with BC as compared to females who reported only their mother affected with BC. The estimated risks were even higher when both mother and sister were affected with BC. Evidence of family history of BC in the first degree relatives and BC risk has been well documented by many studies with different study designs [14, 18]. The variation of reported estimated risks was due to family history nature such as affected age, number and type of the affected family members [19, 20]. It is known that BRCA1 and BRCA2 gene mutations are responsible for this strong association for cases diagnosed at young age [21, 22]. The stronger effect of family history among pre-menopausal females in this study suggested a component of familial BC [20]. Possible explanations to higher estimated risks observed in subjects with sibling affected include recall bias. With self-reported data, maternal history is more likely to be incomplete as compared to the sibling history. Another possibility is the confounder effect such as parity; mothers of subjects were obviously parous while sisters could be either parous or nulliparous. It is known that parity is a protective factor against BC hence if subject’s sisters were null-parous; one would expect to observe higher risk. Sisters are more likely to share the same or similar environmental factors than mother and a daughter. Finally, multiple family relatives having an early onset or bilateral cancer increases the risk even more [15].

### Deprivation score

Deprivation score data was available for the dataset. Our result suggested that the most deprived females appeared to have lower BC risk compared to least deprived females in the UK Biobank cohort. Our cohort appeared to be mainly from least deprived districts like Bristol (8.8%), Leeds (8.9%), Newcastle (7.4%), and Nottingham (6.8%). Most deprived districts included Stockport (0.76%), Manchester (2.7%), and Birmingham (4.9) contributed less in this cohort. This sampling distribution could have an effect on the association direction between deprivation and BC.

### Variables related to body size

Inverse associations were observed with BMI and waist to hip ratio in the pre-menopausal group. While among post-menopausal females, increased risks were reported. A Norwegian prospective study suggested a decreased risk of BC among overweight and obese females who had no family history of BC. Nevertheless once a female has a family history, that protection effect disappeared in both overweight and obese pre-menopausal females [23]. A meta-analysis conducted in 2012 showed no significant effect of BMI on the incidence of pre-menopausal BC [24]. Our results however suggested that risk was reduced even when family history of BC was present among pre-menopausal females. One study reported an estimation of 3% risk increase in BC for every 1 kg/m^2^ in post-menopausal females [25], while another study reported that weight gains of 5–12 kg increases the post-menopausal BC risk by 50% and modest weight loss (5–10%) can decrease BC risk by 25–40% [26]. Furthermore, overweight and obesity are associated with poor prognosis and increased BC mortality [27]. BMI is a modifiable factor and can contribute to reduce the BC risk by 10.0% in pre- and 5.1% in post menopause women [28]. Our study confirmed a BC risk reduction of 8.3% if females reduced their BMI lower than 30 among general population but if obese females (BMI≥30) reduced their BMI to normal BMI range, a 19.4% of BC risk will be eliminated among post-menopaused females. Another way to assess central adiposity among individuals is by measuring WHR (waist to hip ratio). A systematic review on the relationship of WHR and BC concluded that 24% risk reduction was associated with small WHR in post-menopausal females. In contrast among pre-menopausal the effect was very little [29]. Another review suggested the same conclusion; pre-menopausal BC is not associated with WHR however, 1.4 to 5.4 times of BC risk was proven among post-menopausal females [30]. Our study showed BC risk reduction was associated with increased WHR up to 25.6% in pre-menopausal females but failed to prove any association with post-menopausal females. The findings on height and BC risk supported adult height being associated with BC risk in both pre- and post-menopausal groups. The EPIC cohort study [31] reported a positive association between height and post-menopausal BC (RR 1.10 with 95% CI 1.05–1.16). Furthermore, a meta-analysis of 159 prospective studies showed a pooled BC RR of 1.17 (95% CI = 1.15–1.19) per 10cm increase in height [32, 33]. Another pooled analysis also suggested positive association among post-menopausal females (RR = 1.07 with 95% CI: 1.03, 1.12) [34]. No association was reported in pre-menopausal females (RR 1.02 with 95% CI: 0.96, 1.10). Not all prospective studies confirmed the positive association. A register-based cohort study with 13,572 participants concluded no statistical evidence of association between height and BC risk [35]. Evidence from case-control studies was inconsistent. Our study showed an increased risk of 18% per 10cm increase in height among pre-menopausal and 23% per 10cm increase in height among post-menopausal. All the results mentioned previously were for standing height; we examined sitting height and found a BC risk association with sitting height. Taller sitting height is associated with 25.5% BC risk increase per each 10 cm increase in pre- and 37.0% in post-menopausal per 10 cm increase.

The relationship between height and BC suggests a protective effect among females with short stature rather than a continuous increased risk with the increasing of female’s height. One possible explanation is that short females would be exposed to lower levels of insulin like growth factor 1 (IGF 1) throughout childhood and adolescence. IGF-1 is considered to be a strong mitogen for BC cells and IGF-1 receptors are expressed in breast tumour tissues 10 folds higher than normal breast tissues [36, 37].

### Reproductive factors

Our findings suggested protective effect of factors related to childbearing and having more children among pre- and post-menopausal females. Risk factors in pre-menopausal females were early menarche age (<13 years old), late age at first live birth (>25 years of age), high reproductive interval index, and increased duration of OC used were considered as risk factors for BC in pre-menopausal females. Factors such as nulliparous, high reproductive interval index and increased duration of OC used were risk factors in post-menopausal females.

Increased production of steroid hormone starts around the time of menarche and decreases significantly near the menopause [4]. Hormones produced by the ovary directly affect the breast function and development. Studies showed long period of hormonal exposure increases the risk to develop BC. Late menarche and early menopause are known to be protective factors as the period of hormonal exposure is reduced. Lengthening the reproductive years by an early menarche of one year has a stronger effect than delaying the menopause by one year [4]. The strength of menarche age and menopause age on BC development can be affected by BMI [38, 39]. The association between the BC and menopause age can be weaker among post-menopausal females with high BMI as seen in the meta-analysis [4]. Our results showed an evidence of BC risk reduction by late age of menarche but not by early age the menopause age as the previous studies even when BMI was adjusted for in the analysis. A meta-analysis of 120,000 BC cases and 300,000 controls done by a collaborative research group confirmed the existing association between early menarche and developing risk of BC. Extra risk is associated with lengthening female’s reproductive years by one year during menarche rather than lengthening one year at menopause [4]. The RR associated with early menarche was 1.05 (95% CI 1.04–1.06) and the RR associated with late menopause was 1.03(95% CI 1.03–1.03) [4].

Childbearing in a known protective factor against BC although other factors might help confound this protection, such as breast feeding [40]. Combination of both factors can help protect females even more. Unfortunately there were no data available on breastfeeding in our cohort and unable to assess this effect. In the case of parity, our results showed a significant evidence of risk reduction among both pre- and post- menopausal females with a stronger effect among pre-menopausal. Likewise, as the number of children increases, the protective effect increases. Our results suggested an elimination of 9.2% among pre- and 17.9% among post- BC risk associated with being a parous female while other study reported a lower yet an affective risk reduction of 13.3% for the same factor [41]. As the number of children increases, the attributed risk reduction increases accordingly with reduction of 5.2% among pre- and 5.4% among post-menopausal females [28]. Nevertheless, our results suggested a higher reduction among pre- (8.8%) and a lower reduction percentage among post- menopausal women (4.6%).

Termination of pregnancy, whether induced or natural did not appear to affect the BC risk. Thus, younger age at childbirth is a protective factor against BC and this was observed among pre-menopausal females with p values <0.05. Studies showed early pregnancy causes permanent morphological changes to the breast and makes it more resistant to carcinogenic changes [7]. Our study supported the elimination of 44.6% of BC risk if females in general had their first child in their 20s rather than ≥30 years old among pre-menopaused females. This reduction can reach up to 48.4% among females who had their first child at age of ≥30 if they had their first child in their twenties. Furthermore we explored the reproductive interval variable (duration between the menarche and first child) and the results supported evidence reported in the literature that as the duration increases the risk also increases. Long term hormonal exposure has been confirmed to be a risk for BC [15]. Our study showed a BC reduction of 14.9% in pre-menopausal women if they have reproductive interval of < 16 and this reduction can reach up to 29.6% if those females with reproductive interval of ≥16 had interval of 12 or less among pre-menopausal females.

Mammogram history suggested borderline significant increased risk in pre-menopausal women and no association in post-menopausal women. The mammogram itself *per se* is not a risk factor for BC but women who reported having had a mammogram were more likely to be diagnosed. Mammogram screening is proved to reduce the BC mortality by 29% among females aged between 50–69 years [42].

### Hormone use

Oral contraceptive use is known to be a risk factor of BC and this risk rises with longer duration of use [43]. It has been proposed that using OC can activate breast tumours which are already present. Oestrogen is recognized as enhancing tumour growth, and with OC and later HRT use these hormones promotes the tumour growth even more [43]. Our findings suggested a positive association between BC and OC duration amongst pre- menopausal females only. Moreover, HRT users showed 14.1% more risk for developing BC among our cohort. Extensive evidence showed an increase in BC incidence in current HRT users and that risk returns to normal soon after use terminates. Combined oestrogen-progesterone therapy revealed higher risk compared to oestrogen only preparations including results from the Women Health Initiative study (WHI). Recent results from WHI found both oestrogen only and combined formulations convey greater risk for BC if the females started the HRT in less than 5 years after the menopause compared to longer gap [38, 44–47]. The study also carried out further analysis of HRT. Their results showed attenuated BC risk among obese females which is driven by hormonal adiposity of the breast. Endogenous oestrogen rises with the increase of the BMI among HRT non-users which increases the breast adiposity [38]. Another major study carried out in the UK (Million Women Study) identified that BC risk is associated with current use of HRT and the risk is considerably greater among combined oestrogen- progesterone users than other types of HRT [48]. According to our analysis stopping HRT can reduce the risk by 5.8% and by 12.5% risk among HRT users. The Million Woman Study estimated this figure to be 4.6% [41] and a more recent study has put this figure higher at 14.5% [28].

In conclusion, we carried out an analysis to confirm risk and protective factors and BC risk in the UK Biobank female cohort. The findings suggest that protective factors in women included reducing BMI, waist to hip ratio, increasing the numbers of births, having birth at an early age, minimising the use of oral contraceptive and HRT and their durations. Most of our findings are in keeping with evidence reported from the other UK large cohort studies such as the One Million Women and EPIC studies. Evidence from this large study can be further used in translational research such as prevention programmes. Our study has some strengths and limitations. The strengths of this study are: large nation-wide prospective population-based cohort with a follow up time of 9 years and a sizable number of incident cases (UK Biobank). Furthermore, to our knowledge, this is the first study investigating the effect of the anthropometric and reproductive factors with BC risk among the UK Biobank female cohort. The results of this study can be used to inform BC prevention strategies and be used to educate the public and form a basis for building risk prediction models for BC for the UK population. Additionally reproductive interval index is a new measure and only reported by our study using UK data. Estimation of the general PAF and the PAF of the subgroups for BC in the UK Biobank female cohort is novel. The attributable risks calculated for the modifiable factors can be translated into action to reduce BC incidence.

One of the study limitations is that the UK Biobank cohort is not the best representation of UK female population. A recent study investigated the sociodemographic characteristics of the UK biobank participants compared to normal UK population [49] found an evidence of “healthy volunteer” selection bias among the participants. UK biobank participants tend to be healthier, more educated and living in less deprived areas. This effect is common with other volunteer cohorts. Nonetheless, to overcome this limitation and to produce more generalizable associations it is very essential to use large sample size with high internal validity [50, 51]. Our study used a decent sample size and confirmed the expected associations which are similar to the published literature.

Another possible limitation is the lack of information such as breastfeeding history, ovarian cancer family history, BC onset of the family members and BC subtype (PR+, ER+, HER2+, triple negative). Some of the risk factors may affect BC subtype differently [52]. Finally, small sample size in some of the associations such as family history of breast cancer. There were only4 observations among pre-menopaused with both mother and sister family history which can affect the strength of the findings.

## Supporting information

S1 Table Codes used to identify breast cancer cases and controls. (DOCX)

S2 Table Classification of the variables included in the analysis. (DOCX)

S3 Table Summary of the significant factors associated with breast cancer among both pre- and post-menopausal females in the UK. (DOCX)

## Abbreviations

RR: Relative risk
BMI: Body mass index
BC: Breast cancer
CI: Confidence interval
EPIC: The European Prospective Investigation into Cancer and Nutrition
HRT: Hormonal replacement therapy
IGF-1: Insulin-like growth factor 1
ICD: The International Classification of Diseases
IQR: Interquartile range
LCL: Lower confidence limit
NHS: National Health Service
OC: Oral contraceptive
PAF: Population attributable fractions
SD: Standard deviation
UCL: Upper confidence limit
WHR: Waist to hip ratio
