# Supplementary material for: Risk of breast cancer in the UK biobank female cohort and its relationship to anthropometric and reproductive factors
Source: PLoS One. 2018 Jul 26;13(7):e0201097. doi: 10.1371/journal.pone.0201097 (PMC6062099; doi:10.1371/journal.pone.0201097)
Supplement: S1 Table — (DOCX) [file pone.0201097.s001.docx]

S1 Table: Codes used to identify breast cancer cases and controls

| **Categories** | **Frequency (%)** | **ICD10 codes** | **ICD9 codes** | **Self-reported cancer’s codes** | **Self-reported non-cancer diseases** |
| --- | --- | --- | --- | --- | --- |
| **Breast cancer cases** | | | | |  |
| Incident: | 3,378 (1.24%) | Codes start with C50 and its subclasses , C501, C502, C503, C504, C505, C506, C507, C508, and C509 | Codes start with 174 and its subclasses 1741, 1742, 1743, 1744, 1745, 1746, 1747, 1748, and 1749 | 1002 code only |  |
| Prevalent: | 10,853 (3.97%) |  |  |  |  |
| **Subjects excluded from the study** | | | | | |
| 1. Other cancers | 23,540 (8.61%) | Codes start with C except codes for BC | Codes start with 1 or 20 except codes for BC | All other codes except 1002 code |  |
| 1. Breast In situ carcinoma | 636 (0.23%) | Codes of D050, D051, D057, D059 | 2330 code only |  |  |
| 1. Other in situ carcinoma | 2,463 (0.90%) | Codes start with D0 except codes for breast in situ carcinoma | Codes start with 230 or 231 or 232 or 233 or 234 except codes for breast in situ carcinoma |  |  |
| 1. Neoplasm of unknown nature or behavior | 121 (0.04%) | Codes start with D37 or D38 or D39 or D40 or D41 or D42 or D43 or D44 or D45 or D46 or D47 or D48 | Codes start with 235 or 236 or 237 or 238 or 239 |  |  |
| **Controls** | | | | | |
| All controls | 232,476 (85.01%) | Remaining codes or subjects with no code assigned | Remaining codes or subjects with no code assigned |  | All of Self-reported of non-cancerous diseases or no code assigned |
| **Total** | 273,476 (100%) | | | | |
