# Supplementary material for: Risk of breast cancer in the UK biobank female cohort and its relationship to anthropometric and reproductive factors
Source: PLoS One. 2018 Jul 26;13(7):e0201097. doi: 10.1371/journal.pone.0201097 (PMC6062099; doi:10.1371/journal.pone.0201097)
Supplement: S2 Table — (DOCX) [file pone.0201097.s002.docx]

S2 Table: Classification of the variables included in the analysis

|  | Variable | Groups | Coding |
| --- | --- | --- | --- |
| 1 | Menopausal status | Pre-menopausal | Pre- menopausal: reported as pre-menopausal & no history of hysterectomy or bilateral oophorectomy & their age is ≤55 and menarche age ≥7 years old (to make sure we have the maximum number of the real pre-menopausal females - so any female reported as pre- and their age > 55 or had menarche age < 7 and did not had hysterectomy nor oophorectomy will be removed – most probably this female is miscategorised). |
|  |  | Post-menopausal | Post-menopausal: reported as post-menopausal & no history of hysterectomy or bilateral oophorectomy (only natural menopause) & their menopause age is ≥ 40years old (to make sure we have the maximum number of the real post-menopausal females – so any female reported as post- and their menopause age < 40 and did not had hysterectomy nor oophorectomy will be removed - most probably this female is miscategorised). |
| 2 | Menarche age | (>13) | This variable was divided into two groups based on literature ranges [4]. |
|  |  | (≤13)) |  |
| 3 | Age at first birth | (<20) | This variable was divided into four groups based on literature ranges [7]. |
|  |  | (20-24) |  |
|  |  | (25-29) |  |
|  |  | (≥30) |  |
| 4 | BMI | Healthy (18.5 – 24.9) | This variable was divided into three groups based on WHO classification [10]. Underweight group were very low in number and wouldn’t be enough for the association calculations. |
|  |  | Overweight (25-29.9) |  |
|  |  | Obese (≥30) |  |
| 5 | Waist to hip ratio (WHR) | Low (≤0.80) | This variable was calculated by dividing the waist over the hip measurements of the participants. Then later was divided into three groups based on WHO classification [11]. |
|  |  | Moderate (0.81-0.85) |  |
|  |  | High (>0.85) |  |
| 6 | Reproductive interval index- years | Low (≤12) | Reproductive interval index is the difference between the age at first birth and age at menarche. The index was divided into four groups based on the IQR (InterQuartile Range) of the reproductive interval index values among the controls only. |
|  |  | Moderate (12-16) |  |
|  |  | High (>16) |  |
|  |  | No children |  |
| 7 | Deprivation score | Calculated by UK biobank team | The score was calculated for all the participants before participating in UK Biobank. The score was based on the prior national census output areas [12]. The score evaluates four aspects: 1) unemployment, 2) houses without an owned car, 3) non-house ownership, 4) overcrowding in one house [13]. Data were provided by the UK biobank team. |
| 8 | Height | Below mean (< 156.10 cm) | The height was grouped into three groups based on the mean of the control group. |
|  |  | Within mean ± SD (156.10-168.75cm) |  |
|  |  | Above mean  (>168.75 cm) |  |
