# Supplementary material for: Risk of breast cancer in the UK biobank female cohort and its relationship to anthropometric and reproductive factors
Source: PLoS One. 2018 Jul 26;13(7):e0201097. doi: 10.1371/journal.pone.0201097 (PMC6062099; doi:10.1371/journal.pone.0201097)
Supplement: S3 Table — (DOCX) [file pone.0201097.s003.docx]

S3 Table: Summary of the significant factors associated with breast cancer among both pre- and post-menopausal females in the UK

| **Variable** | **Pre-menopausal (effect size)*** | **Post-menopausal (effect size)*** | **Conclusion** | **Modifiable** |
| --- | --- | --- | --- | --- |
| **Non-modifiable** | | | | |
| Age (continuous) | Risk (5%) | Risk (3%) | Getting older – more risk | No |
| BC family history (categorical) | Risk (77%) | Risk (58%) | Family history – more risk | No |
| Deprivation score (continuous) | Protective (3.8%) | Protective (2.7%) | More deprived – less risk | Yes |
| Sitting Height in cm (continuous) | Risk (2.3%) | Risk (3.2%) | Taller - more risk | No |
| Standing Height in cm (continuous) | Risk (1.7%) | Risk (2.1%) | Taller - more risk | No |
| Standing Height in cm (categorical) | Risk (42.9%) | Risk (53.3%) | Taller - more risk | No |
| Menarche age in years (continuous) | Protective (5.2%) |  | Older – less risk | No |
| Menarche age (categorical ) | Protective (22.8%) |  | Older – less risk | No |
| **Modifiable** | | | | |
| BMI (continuous) | Protective (1.7%) | Risk (1.8%) | High BMI – less risk in pre- and more risk in post | Yes |
| BMI (categorical) | Protective (26.7%) | Risk (24.1%) | High BMI – less risk in pre- and more risk in post | Yes |
| Waist Circumference in cm (continuous) | Protective (0.8%) | Risk (0.8%) | High waist circumference – less risk in pre- and more risk in post | Yes |
| Hip Circumference in cm (continuous) |  | Risk (1.2%) | High hip circumference – more risk in post | Yes |
| Waist to Hip (continuous) | Protective (86.9%) |  | High ratio – less risk in pre | Yes |
| Waist to Hip (categorical ) | Protective (25.6%) |  | High ratio – less risk in pre | Yes |
| Contraceptive duration in years (continuous) | Risk (2.4%) |  | Larger interval – more risk in pre | Yes |
| HRT use (categorical) |  | Risk (14.1%) | HRT use – more risk I post | Yes |
| **Partially modifiable** | | | | |
| Parity (categorical) | Protective (23.6%) | Protective (17.9%) | More children – less risk | Yes |
| Number of births (continuous) | Protective (7.5%) | Protective (10.1%) | More children – less risk | Yes |
| First live birth age (categorical) | Risk (93.8%) |  | Older – more risk | Yes |
| Reproductive interval index in years (continuous) | Risk (0.3%) | Risk (0.3%) | Larger interval – more risk | Yes |
| Reproductive interval index (categorical) | Risk (53%) | Risk (33%) | Larger interval – more risk | Yes |
